# Three-Month Durability of Bilateral Two-Level Stellate Ganglion Blocks in Patients with Generalized Anxiety Disorder: A Retrospective Analysis

**DOI:** 10.3390/brainsci15020188

**Published:** 2025-02-13

**Authors:** Sean W. Mulvaney, Kyle J. Dineen, Sanjay Mahadevan, Roosevelt Desronvilles, Kristine L. Rae Olmsted

**Affiliations:** 1 Department of Military and Emergency Medicine, Uniformed Services University, 4301 Jones Bridge Road, Bethesda, MD 20814, USA; seanmulvaney@hotmail.com; 2 Orthobiologics Research Initiative Inc., 11200 Rockville Pike #230, North Bethesda, MD 20852, USA; smahade3@alumni.jh.edu (S.M.); roosevelt@rosm.org (R.D.J.); 3 RTI International, 3040 E Cornwallis Rd., Research Triangle Park, NC 27709, USA; krolmsted@rti.org

**Keywords:** stellate ganglion, SGB, anxiety, generalized anxiety disorder, GAD-7, two-level cervical sympathetic chain block, 2LCSB, ultrasound

## Abstract

**Purpose:** Determine if performing ultrasound-guided, bilateral, two-level cervical sympathetic chain blocks (2LCSB) (performed on subsequent days) provides durable improvement in symptoms associated with anxiety. **Methods:** A retrospective chart review was conducted between January 2022 and November 2024. We identified 114 patients who received bilateral, 2LCSB for anxiety symptoms. Generalized Anxiety Disorder 7-Item Scale (GAD-7) outcome measure scores were collected at baseline and three-months post procedure in 71 males and 43 females. **Results**: Out of 114 patients, 99 patients (86.8%) showed a long-lasting improvement in their GAD-7 scores. Collected GAD-7 forms had a baseline average of 15.52 (14.99 for males and 16.40 for females), which decreased after three months to an average of 7.28 (6.96 for males and 7.81 for females). This represents a 52% average improvement in anxiety symptoms. **Conclusions:** In individuals treated with bilateral, 2LCSB, GAD-related symptoms were improved by 52% for at least 3 months regardless of initial anxiety severity.

## 1. Introduction

Anxiety disorders (ADs) are a leading cause of psychological stress globally, with a high burden of illness for many patients [1,2]. There are over 301 million globally recorded cases of anxiety as of 2019, with an additional projected 25% increase in overall cases following the COVID-19 pandemic [3,4]. ADs may present with unique symptoms based on the specific diagnosis, but are symptomatically described as excessively perseverating on worries, fears, concerns, or aspects of daily living. This may result in challenges related to employment, personal relationships, management of finances, and other aspects of life [5]. ADs are typically chronic and comorbid with other psychological health conditions such as post-traumatic stress disorder (PTSD), substance abuse disorder, depression, phobic conditions, and suicidal ideations [5] ADs are diagnosed more frequently in females than males, with females having a 1.5–2 times greater likelihood per Bendelow et al. [2]. Their specific etiology is unknown but is thought to be due from a combination of childhood trauma, stressful life circumstances, genetic differences in patients, and other unknown causes which manifest in both neurocognitive and neurobiological challenges [6,7].

Traditionally, anxiety disorders are treated through a combination of behavioral therapy and psychopharmacological intervention. Behavioral therapy is shown to provide notable benefits in patients with anxiety, with roughly 50% improvement with cognitive behavioral therapy (CBT) [8]. Common psychopharmacological interventions include serotonin reuptake inhibitors (SSRIs), serotonin–norepinephrine reuptake inhibitors (SNRIs), anti-psychotics, GABAergic medications, and anti-depressants, among other options [1]. While at times useful, anti-anxiety medications may also be over-prescribed and have various common side effects from their intervention [9]. These include drug dependence, oversedation, and impact on neurocognitive functions, which may affect interpersonal relationships and overall psychological health [9], sought in cases of pervasive anxiety-related symptoms. The published literature notes between 60 and 85% of patients respond to anxiety-specific medications and only 50% experience symptom relief [10]. Anxiety may be refractory, as patients experience high rates of recurrence following treatment [11], with lifestyle factors, comorbid conditions, and poor psychopharmacological responses often contributing to return of symptoms [12].

Unfortunately, over the past ten years, there have been significantly less clinical trials aimed at providing new psychopharmacological options for patients with anxiety disorders relative to other psychiatric conditions [9]. Procedural options for management and the treatment of anxiety are also relatively scant. Deep brain stimulation (DBS) has been explored for a range of psychiatric conditions with mixed results. Some pre-clinical studies on mice suggest that DBS of the hypothalamus may provide therapeutic improvements [13], but case-series-level evidence in humans found increased panic and fear with DBS in a 2006 publication [14]. Other works suggest that DBS may have future applications on psychiatric conditions, but anxiety-specific applications were not explicitly listed [15]. Cranial electrotherapy stimulation (CES) is another procedural option proposed to treat clinical disorders. In a small number of pre-clinical and clinical studies, some publications showed limited initial benefits of CES but with conflicting results and severe methodological concerns, bias, and lack of scientific rigor [16,17,18]. When traditional therapeutic and pharmacological measures fail, patients with anxiety may struggle to find alternative treatment options. For this reason, it is critical to continue conducting research trials focused on providing new treatment options for this disorder [1].

Patients often report anxiety-related symptoms of hyperarousal, anxiousness, and irritability, which may result from the chronic activation of the sympathetic nervous system (SNS). Heightened activation of the SNS is essential for surviving danger during immediate fight-or-flight scenarios but may remain inappropriately elevated in patients with chronic anxiety. The stellate ganglion is a part of the cervical sympathetic chain, along both sides of the anterior cervical spine, and is a cluster of nerves located in proximity to the C7 vertebra that results from the fusion of the inferior cervical and first thoracic sympathetic ganglia. It is thought to play a significant role in the mediation of the sympathetic nervous system and may mediate communication between the body and the brain along the SNS, and blocking the stellate ganglion may assist in reducing anxiety-related symptoms [19].

The stellate ganglion block (SGB) procedure is an emerging procedure option shown to improve anxiety symptoms in clinical settings. The intervention was first used in the 1920s for the treatment of pain-related conditions and post-traumatic stress injury. In 1990, Lebovits et al. noted and described the first use of SGB for the treatment of post-traumatic stress disorder (PTSD), which is often comorbid with anxiety [20]. Since this 1990 publication, there have been more than thirty-five peer-reviewed studies using SGB as a treatment modality for PTSD and anxiety [21]. Most notably, SGBs have been shown to be a safe, well-tolerated treatment option for PTSD symptoms, with Level 1b evidence affirming its use.

Since the 1990s, there has been significant evolution regarding the methodology of the SGB procedure. The development of injection protocols was heavily influenced by an understanding of the central and autonomic nervous system stress responses. SGBs were initially conducted by injecting the right side at the 6th cervical vertebra level [22,23], as the right cerebral hemisphere impacts the central and autonomic nervous system’s stress response [24,25]. However, future works found two-level cervical sympathetic chain blocks had superior improvements in PTSD symptoms when conducted at the 4th and 6th cervical vertebrae in comparison to single-level SGB at the 6th cervical vertebra alone [26,27]. In 2019, Rae Olmsted and colleagues published their landmark Level 1b multi-center randomized controlled trial (RCT), which demonstrated the efficacy of right-sided SGBs (injecting 7–10 mL of 0.5% ropivacaine) spaced two weeks apart for the treatment of PTSD-related symptoms [28]. As a result, SGB was denoted as an available treatment option that can be used in conjunction with trauma-focused psychotherapy and has been incorporated into behavioral centers over the last 10 years [29].

Since 2015, published Clinical Practice Guidelines described the use of left-sided SGB for PTSD if right-sided SGB did not provide clinical benefits [30]. In 2021, the first case series illustrated that 5% of patients do not respond to right-sided SGB and will only respond to left-sided SGB treatment for PTSD. Before this, SGB-related benefits were only described with right-sided blocks in the research for PTSD treatment [31]. As a result, we shifted our clinical practice and data collection to reflect the usage of a bilateral, multilevel block for PTSD treatment. To prevent occult medical emergency caused by recurrent laryngeal nerve block and subsequent potential respiratory distress, SGBs were performed on subsequent days. Patients receive the right-sided block first, then the left-sided block on the following day.

The term SGB, and its corresponding procedure, are well known, but it should not be considered accurate nomenclature for the intervention in our view. Patients present with significant anatomical variations in the course of the cervical sympathetic chain, and performing the block at the 6th and 4th cervical vertebrae levels resulted in improved outcomes versus the C6 level alone [19,32,33]. The stellate ganglion is also only present in 80% of individuals—therefore, the term SGB may present an inaccurate assessment of the procedure [34]. For this reason, we began using two-level cervical sympathetic chain block (2LCSB) as the more appropriate medical nomenclature for the SGB procedure, since it describes treating the cervical sympathetic chain, and not specifically the stellate ganglion.

While using bilateral 2LCSBs to treat PTSD in clinical practice, we noticed secondary patient complaints were improving during follow-up. While all patients underwent treatment for PTSD symptoms, we observed significant improvements in secondary, overlapping symptoms (such as anxiousness, worry, and restlessness) in patients with a comorbid history of anxiety. This observation prompted us to include patient-reported outcome measures (PROMs) for anxiety-specific symptoms using the Generalized Anxiety Disorder 7-Item Scale (GAD-7) questionnaire [35].

In terms of anxiety-specific application of the 2LCSB, Lynch and colleagues published the first case series suggesting SGB was an effective treatment option for anxiety symptoms [36]. Using the GAD-7 as the primary metric, this study showed bilateral 2LCSBs to be a safe and possibly effective intervention for chronic severe anxiety. Changes in patient GAD-7 scores at one week showed a 57% improvement in anxiety symptoms and GAD-7 scores at one month showed a 48% improvement. A significant observation in that study noted that bilateral 2LCSB demonstrated more improvements than a lone right-sided procedure [36]. It also noted that some patients responded to repeat right-sided 2LCSB treatment if symptoms recurred after undergoing bilateral 2LCSB sequence [37]. The Lynch et al. publication [36] followed patients for only 1 month after treatment. While the study showed a significant reduction in symptoms for severe anxiety in patients regardless of sex/gender, effects in individuals with mild or moderate symptoms remain undescribed. The primary goal of this publication is to investigate these outstanding questions by increasing follow-up to three months post procedure, including secondary goals of analyzing effects in patients diagnosed with mild to severe anxiety, conducting an analysis of subgroup-specific responses, and modeling male and female sex outcomes after the 2LCSB procedure. We hypothesize that 2LCSB may provide a long-lasting improvement in GAD-7 score decreases at three months post procedure across all severity groups and for male and female patients.

## 2. Materials and Methods

This retrospective cohort case series was approved by the institutional review board of the Institute of Regenerative and Cellular Medicine (IRCM-2024-412). Patients (n = 114) with a prior history of chronic anxiety, documented for greater than 3 months, were selected as participants. In each case, patients underwent a bilateral 2LCSB procedure between January 2022 and September 2024 at the Regenerative Orthopedics and Sports Medicine (ROSM) clinic in Annapolis, Maryland. Patients were included in this review based on the following anxiety diagnosis criteria: a behavioral health provider’s formal diagnosis and a score of 5 or greater on the GAD-7 questionnaire. Comorbid health conditions were not an exclusionary factor, as all patients were seen as a part of regular clinical practice and were seeking interventional aid for their persistent anxiety symptoms. Additionally, there was no randomization into placebo and interventional groups since all participants were clinical practice patients. Patients with a minimally anxious diagnosis per the GAD-7 (scoring 0–4) were excluded from the cohort, as they did not meet a sufficient degree of symptom presentation.

The GAD-7 is a seven-item self-reporting instrument used to assess symptoms of anxiety disorders. It contains seven questions, each scored on a scale of 0 to 3, with a range of 0 to 21. Table 1 reflects the questions provided in the form and the corresponding scoring system. It is easily administered and maintains 68% sensitivity (89% for generalized anxiety disorder) and 88% specificity for common anxiety disorders [5]. A cumulative score of 5–9 indicates mild anxiety, 10–14 indicates moderate anxiety, while 15 or greater indicates severe anxiety [5]. All patients completed PROMs in the form of the GAD-7 pre-2LCSB procedure (denoted as baseline GAD-7 or BL GAD-7) and three-months post-2LCSB procedure (denoted as 3M GAD-7). The GAD-7 was provided to patients through a cloud-based, secure data-collection system, with 21 CFR Part 11 data compliance, at baseline and three-months post procedure. Scores were entered into the secure data-collection system that is only accessible by the principal investigator and clinical research staff to reduce bias or exposure of protected health information. The physician that performed all 2LCSBs did not analyze any collected data until IRB approval was received. There was no formal study enrollment period, as patients were treated as a normal part of our clinical practice.

Cervical sympathetic blocks are considered a well-tolerated and safe procedure, with only five case reports indicating patient complication [38,39,40,41]. All 2LCSB’s performed in this study follow the strict and rigid guidelines provided affirmed by publications produced in the last three years. The following text provides a detailed description of the 2LCSB performed on each patient according to our procedure protocol.

“Per published guidelines, all patients had a bilateral 2LCSB at the 4th cervical vertebra and 6th cervical vertebra, with the right side performed on day 1, and the left side performed on day 2 [23]. The blocks were performed on subsequent days to eliminate associated potential airway compromise. A Doppler ultrasound scan was utilized prior to every procedure to clearly identify the vertebral artery and vein, as well as other vasculature in accordance with published literature. The use of ultrasound imaging improves visualization of target structures, minimizing risk of damage to soft tissue, vascular, and nerve anatomy [42,43]. A 50-mm 25-gauge needle was utilized under ultrasound-guidance (General Electric Logic e with an 8–12 MHz broadband linear transducer) using a lateral, in-plane approach at both the 6th cervical vertebra level (using 6–8 mL of 0.5% ropivacaine) and the 4th cervical vertebra level (using 1.5–2 mL of 0.5% ropivacaine). This dose has been considered standard protocol across many studies and is widely used in clinical medicine [34]. The use of 6–8 mL of ropivacaine has been shown to be well tolerated in published RCTs and systematic reviews, without serious complications reported [44,45]. All procedures were performed at an established musculoskeletal practice by a single anaesthesia/pain medicine and sports medicine fellowship-trained physician who has performed more than 4500 cervical sympathetic blocks. Horner’s syndrome, a sign of a successful blockade of the stellate ganglion, is characterized by objective findings of ptosis, miosis, and scleral injection and was scored by two independent observers 5 min post-block per published guidelines [22]. All patients met the minimum clinical threshold for an acceptable Horner’s syndrome of 4 out of 6 points by both observers. In all cases, no adverse events or complications were reported by patient post-procedure”.

We then conducted a retrospective analysis of the GAD-7 scores to determine changes in patient outcomes. Patients were grouped by a severity-based diagnosis of either mild (scoring 5–9), moderate (scoring 10–14), or severe (scoring >15) anxiety on the GAD-7 questionnaire at baseline (pre-procedure). Scores for each patient were collected and denoted as BL (pre-procedure) and 3M (three months post-procedure). The outcomes were separated to determine sex-based differences for each subgroup and means for mild, moderate, and severe anxiety were assessed. Additional analysis was conducted to determine the distribution of age for each severity subgroup (mild, moderate, and severe, and average).

**Figure 1:**
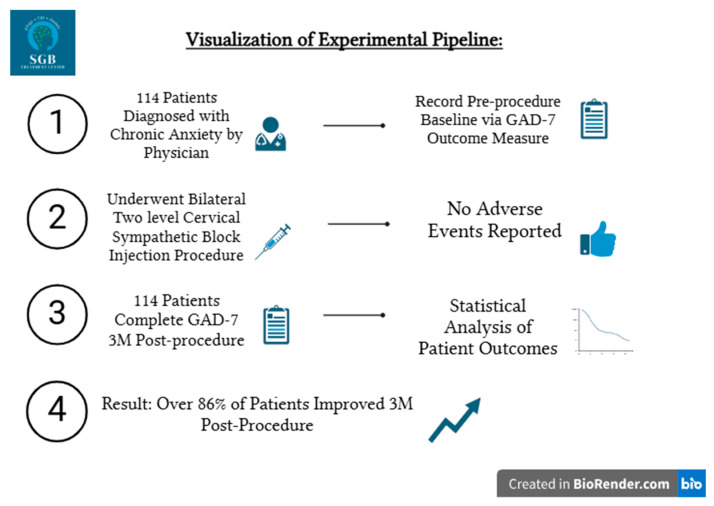
Experimental pipeline used to complete patient enrollment, treatment, and analysis.

Scores were analyzed using repeated-measures ANOVA to compare the mean score of the same subject across the BL and 3M observations. Selected metric variables for this analysis included BL mild, BL moderate, BL severe, 3M mild, 3M moderate, and 3M severe GAD-7 scores. Age and sex were selected as nominal variables for repeated measures ANOVA analysis. Additionally, a T-Test for paired samples (BL and 3M GAD-7) was conducted with the null hypothesis that there is no difference in the mean value between the variables GAD7 Baseline and GAD7 3M, and an alternative hypothesis that there is a difference in the mean value between the variables GAD7 Baseline and GAD7 3M. Data set minimums and maximums, interquartile ranges, confidence intervals (established at 95%), and mean with standard deviation per subgroup were calculated for the collected GAD-7 scores. All results were modeled as raincloud or box plots with confidence intervals, interquartile range boxes, or jiggering to represent distribution of samples in each grouping category. The methodological protocol of this study is shown in Figure 1.

## 3. Results

**Figure 2:**
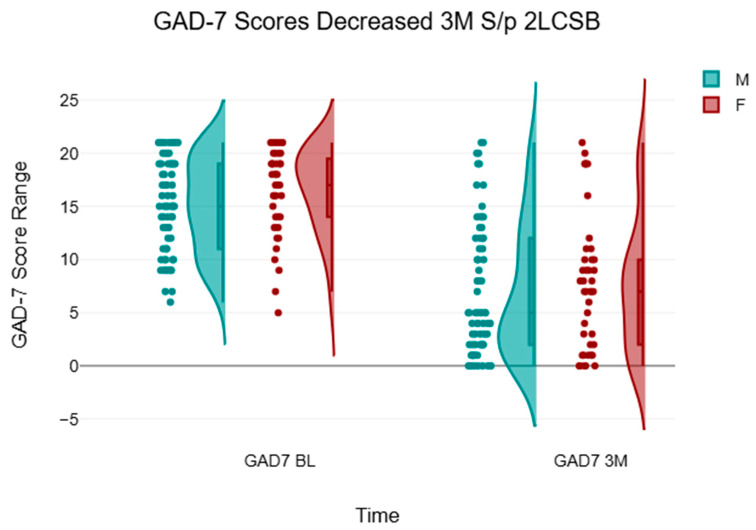
Decrease in GAD-7 scores after three months across all patients independent of severity and isolated by male (M) and female (F). Data modeled using raincloud plot with jiggering.

Out of the 114 patients in this analysis, 99 (86.8%) treated with bilateral 2LCSB in our retrospective analysis saw a decrease in GAD-7 scores and corresponding improvement in their GAD symptoms. A total of 15 patients (13.15%) did not improve at three-month follow-up. The average age for included patients was 47.97 (47.49 for male patients, and 48.77 for female patients) and our data set included 71 men and 43 women. The average GAD-7 baseline score was 15.52 (14.99 for males and 16.40 for females). The average GAD-7 score at three months was 7.28 (6.96 for males and 7.81 for females). These changes are seen in Figure 2.

**Figure 3:**
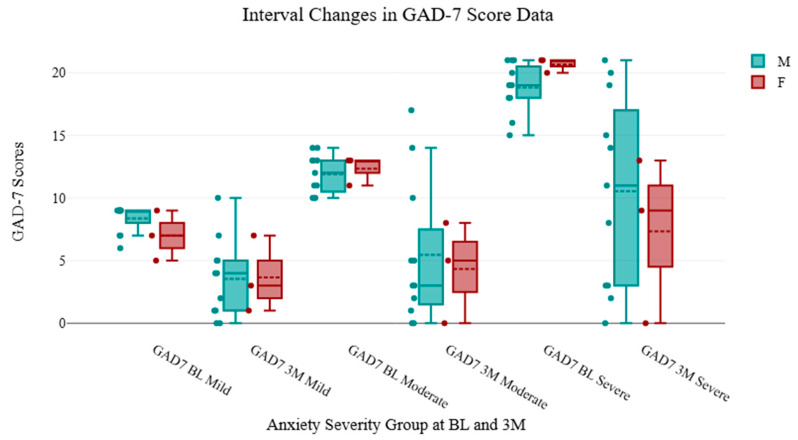
Severity-specific analysis of patient outcomes shows decrease in diagnosis categories 3M post procedure. Baseline (BL) and three-month (3M) scores are visualized across mild, moderate, and severe severity subgroups. This is seen across all severity groups with at least 50% improvement in anxiety-related symptoms. The figure includes a boxplot with CI, data jiggering, and the graphing of male and female data.

**Figure 4:**
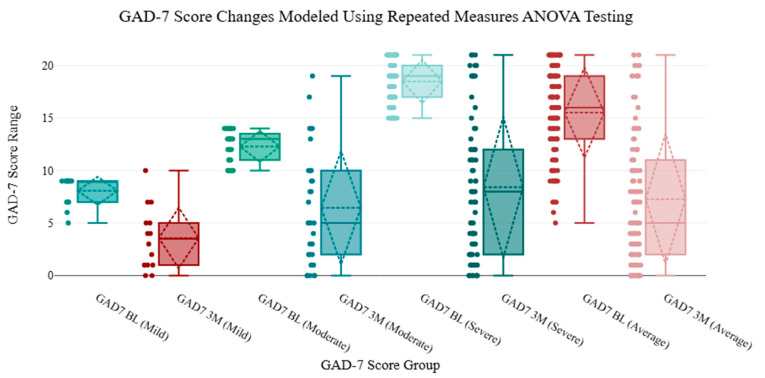
Boxplots with IQR and CI with jiggering to model repeated-measures ANOVA testing for BL and 3M GAD-7. Baseline (BL) and three-month (3M) scores are visualized across Mild, Moderate, Severe, and Average severity subgroups.

**Figure 5:**
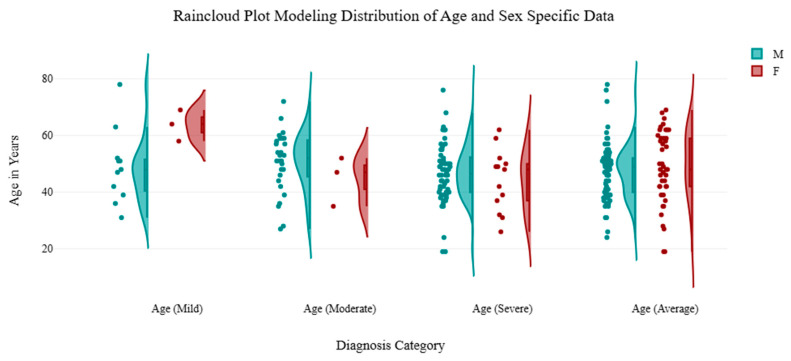
Raincloud plot modeling distribution of age with jiggering for each severity subgroup (mild, moderate, severe, and overall average). Sex-specific variables (male and female patients) are included in the data modeling.

For male patients, mild (5–9), moderate (10–14), and severe (>15) scores at baseline were 8.36, 12.10, and 18.25, respectively, and 3.55, 6.20, and 8.28 at three months. For female patients, mild (5–9), moderate (10–14), and severe (>15) scores at baseline were 7.00, 12.64, and 18.79, respectively, and 3.67, 6.91, and 8.59 at three months. These outcomes are found in Table 2 with percentage changes. The GAD-7 scores across these 114 patients revealed similar decreases in raw score and percent improvement from baseline with durable improvements seen through three-months. These changes are seen in Figure 3 and Figure 4. Using repeated measures ANOVA analysis, all subgroups showed a decrease in GAD-7 scores from BL to 3M. Table 2, Table 3 and Table 4 reflect demographic analysis including subgroup outcomes for anxiety severity (using repeated-measures ANOVA), age-specific ranges for patients, and GAD-7 score analysis. These tables include the F factors for ANOVA measures, degrees of freedom (df), interquartile ranges (IQR), and confidence intervals (CI). Figure 5 graphically models the age distribution for patients included in the trial, including sex and severity group. A review of the retrospective data points to the rejection of the null hypothesis, as this analysis supports the alternative hypothesis that there is a difference in the mean value between the variables “GAD-7 Baseline” and “GAD-7 3M”. F factors for the severe and average groups produced the highest values relative to the mild and moderate categories. Table 5 includes age-specific demographic information for patients included in the cohort review.

## 4. Discussion

As previously discussed, there were three distinct aims of this study. The primary aim was to determine if 2LCSB would provide durable improvements in symptoms at three-month follow-up. The second was to evaluate symptom responses by degree of severity. The third was to investigate sex-specific factors associated with GAD-7 score changes. To our knowledge, this work is the first to show continued improvement in anxiety-related symptoms for up to three months, suggesting some degree of durability associated with 2LCSB. The other difference between this work and the Lynch et al., 2023 study is the use of universal bilateral blocks versus single blocks [36]. All patients received bilateral blocks in this data set, while 70 of the 285 patients received a one-sided block in our previous 2023 study.

Severity-based and sex-based outcomes from the 2LCSB procedure were included in this study, providing subgroup-specific outcomes based on the degree of anxiety diagnosis for both sexes. As an instrument, the GAD-7 incorporates a symptom-scoring system that allows clinicians to grade the degree or severity of a patient’s anxiety with 89% sensitivity and 88% specificity for anxiety disorders [5]. This work shows an initial indication of durable improvements in anxiety-related symptoms for mild (52% improvement), moderate (49% improvement), and severe anxiety (55%) at three months. Our findings include additional insights that both male and female patients with mild, moderate, and severe anxiety all had clinically significant improvements in their symptoms, as shown in Figure 3 and Figure 4. However, due to the lower numbers of patients enrolled into the mild and moderate categories, and given that this was a secondary aim of this analysis, these findings should be interpreted cautiously with a need for higher enrollment in future works.

Anxiety disorders are thought to arise from persistent stress responses [46]. Long-term exposure to stressors prompts dominance of the SNS, which shifts from enabling short-term adaptation towards maladaptive disorder [47,48]. During a sustained chronic stress response, neuroendocrine, autonomic, and behavioral components of biological systems activate, even if threats are only perceived by the individual [49]. Additionally, stress identification varies between individuals but the perception of SNS activation, whether for a real or imagined threat, may also reinforce stress response [50,51]. This increase in allostatic load (chronic stress) is correlated with many psychiatric disorders, including anxiety [52]. The published literature supports this, indicating that chronic mental stress affects sympathetic activity and is associated with issues such as metabolic and cardiovascular disorders [53].

Chronic anxiety also alters brain activity and function through changes in neuroinflammation and neurobiological mechanisms (both oxidative and excitotoxic) which may disrupt an affected patient’s neural circuitry [54]. It is also thought that this disruption may impact proper functioning of the fronto-cingulo-parietal cognitive control network [24]. When affected, a patient may experience improper memory, attention, persistent fear or fear processing, and other cognitive challenges associated with neurological changes [55]. High allostatic load impacts many biological systems, with associated pathology of the hypothalamic–pituitary–adrenal axis [56], hyperactivity of the SNS, immune system dysfunction, and dysfunction of the parasympathetic nervous system (PNS) [57], which is known to increase pro-inflammatory neurotoxic cytokines and neuroinflammation [58,59]. Chronic microglial activation is commonly associated with neuroinflammation [60], as these cells signal monocytes [61] to the brain, which release pro-neuroinflammatory cytokines [62]. This may be a target of study in future works regarding 2LCSB. Lastly, the superior cervical ganglia are also associated with cardiovascular innervation and support proper homeostasis when properly functional, which may support the notion that cervical sympathetic dysfunction is correlated with cardiovascular pathology [63,64]. In short, anxiety disorders are strongly associated with neuroinflammatory patterns and toxin production in the brain [65], among other biological issues.

There is no definitive understanding of the neurobiological mechanism associated with 2LCSB for the observed improvements in anxiety-related symptoms. One predominant theory suggests that 2LCSB prompts durable changes in neuroinflammation, nerve-related immune function, and perfusion changes which may contribute to improved symptoms. Some studies suggest that altering brain inflammatory factors such as NF-kB, along with the perfusion patterns through the cerebral hemisphere, and regulating possible nerve–endocrine–immune system dysfunctions may all play a role in symptom mediation [66,67,68]. Since anxiety disorders are associated with immune, endocrine, and SNS dysfunction, it is reasonable for future research to explicitly investigate related markers in a dedicated mechanistic study, which was not possible in this retrospective cohort review. This may also explain the immediate improvement in symptoms following 2LCSB, as it is thought to modulate neuroinflammation and positively affect the fronto-cingulo-parietal cognitive control network. However, the current supporting evidence is non-specific and requires future scientific research efforts. The lack of a scientific understanding of the mechanism of action is a known limitation and, considering the numerous publications using SGB and related SNS blocks, there remains a need for high rigor studies to investigate 2LCSB’s underlying mechanism.

The authors of this work acknowledge the clear limitations associated with our retrospective cohort review. One challenge in assessing the efficacy of the 2LCSB on anxiety is the lack of a control group to determine change in symptoms, which limits the possible conclusions of the study. Our patient cohort outcomes present as relatively large due to the n number, but a placebo group is needed to determine outcomes more appropriately. This was not possible for the purposes of this study, as included patients were treated within our clinical practice for chronic anxiety. Many patients included in this study also have multiple complex behavioral health issues which were not screened out; therefore, a formal randomized controlled trial with exclusionary criteria is needed to determine generalizability and efficacy of these initial findings. This study did not determine how many patients received adjunctive care external to the 2LCSB, such as behavior health or pharmacological therapies, which may impact the response to the procedure. The relationship between cervical sympathetic blocks with associated conservative care options should also be explored in future works. We also acknowledge that patient symptoms may improve from receiving any baseline treatment, since placebo effects are known to be higher with injection procedures and may alter patient reporting [69]. However, this study, associated with the response to a placebo, investigated subcutaneous injection versus oral delivery of migraine medication, which may not be generalizable to sympathetic blocks with observable Horner’s syndrome. The lack of blinding, a retrospective cohort design, and limited enrolment criteria may all contribute to the bias of the investigators and should be noted. Finally, the use of a single keyer for data entry and analysis may call these data into question.

These findings, in the authors’ view, have some utility in being shared with a wider academic audience. The medical literature now shows improved symptoms from 2LCSB during short- (one week and one month) and longer-term (three month) follow-up, which builds on prior works showing improvement in patient symptoms after one month [15,18,26,28,30,31,36,70,71]. On this topic, this work adds additional evidence of 2LCSB providing anxiety-symptom-relieving outcomes, along with longer follow-up data and severity range information. While these findings should be interpreted with caution considering limitations, over 86% of patients declared post-procedure improvements. Given that approximately 13% of patients failed to report GAD-related improvements from the procedure, further research should be conducted into these non-responders in future studies. We hypothesize that comorbid neuropsychiatric disorders or patient-specific circumstances might contribute to these individuals failing to receive any improvement from the procedure. Given that individuals from both the “male” and “female” groups as well as the “mild”, “moderate”, and “severe” anxiety groups did not improve, this would seem to rule out the possibility of the procedure only providing improvements based on severity or gender. As previously discussed, anxiety disorders remain challenging to treat, and many patients do not report improvements with the initial course of treatment. This real-world cohort, comprising patients with complex medical histories, provides initial indication that cervical sympathetic blocks may offer an alternative option for the treatment of anxiety disorders. 2LCSB is not a first-line treatment and should be used in combination with psychopharmacological and psychotherapeutic interventions [35]. However, we do propose that 2LCSB might improve therapeutic outcomes due to reduced hypervigilance and SNS hyperactivity following the procedure. As we have previously suggested, the chronically heightened activity of the SNS is essential to avoiding danger and surviving high stress circumstances, but SNS dysfunction and hyperactivity may contribute to persistent symptoms of anxiety. By blocking the cervical sympathetic chain with a long-acting aesthetic, the 2LCSB procedure may disrupt the pathological communication of danger between the peripheral and central nervous system [36] after 2LCSB [72,73,74]. This lower SNS activity may potentially enable patients to make greater improvements with conservative care. We recommend that future studies consider how 2LCSB supports patients with and without first-line treatment regiments, which has not been reported on previously.

## 5. Conclusions

In this limited retrospective case series, the use of bilateral ultrasound-guided 2LCSB resulted in an improvement in GAD-7 scores three months post procedure. Patients experienced these improvements in GAD-related symptoms, regardless of severity or gender, with 86% of patients experiencing long-lasting improvement through three months. Further studies, preferably randomized placebo-controlled trials, are needed to assess this procedure for the treatment of anxiety.

## Figures and Tables

**Table 1:** Generalized Anxiety Disorder 7-Item Scale questionnaire.

Over the Last Month, How Often Have You Been Bothered by the Following Problems?	Not at All Sure	Several Days	Over Half the Days	Nearly Every Day
1. Feeling nervous, anxious, or on edge	O	O	O	O
2. Not being able to stop or control worrying	O	O	O	O
3. Worrying too much about different things	O	O	O	O
4. Trouble relaxing	O	O	O	O
5. Being so restless that it is hard to sit still	O	O	O	O
6. Becoming easily annoyed or irritable	O	O	O	O
7. Feeling afraid as if something awful might happen	O	O	O	O

**Table 2:** GAD-7 average baseline and 3-month outcomes with percentage of improvement. A summary of patient outcomes as determined by percent improvement and GAD-7 score improvements. Data are separated by male and female mild, moderate, and severe anxiety, along with general mild, moderate, severe, and total anxiety.

Anxiety Severity (Mild, Moderate, Severe)	GAD-7 BL Mean Score	GAD-7 3M Mean Score	Percentage Change in GAD-7 Scores (%)
Mild Anxiety (Female)	7.00	3.67	37%
Mild Anxiety (Male)	8.36	3.55	56%
Moderate Anxiety (Female)	12.64	6.91	46%
Moderate Anxiety (Male)	12.10	6.20	51%
Severe Anxiety (Female)	18.79	8.59	54%
Severe Anxiety (Male)	18.25	8.28	56%
Mild Anxiety (General)	8.07	3.57	52%
Moderate Anxiety (General)	12.29	6.45	49%
Severe Anxiety (General)	18.48	8.41	55%
Total Average Anxiety	15.52	7.28	53%

**Table 3:** Comprehensive analysis of repeated-measures ANOVA testing outcomes. There is an inclusion of the F factors, degrees of freedom (df), and other statistical variables for each subgroup.

	Sum of Squares	df	Mean Square	F	*p*	η^2^	η^2^_p_
GAD7 BL (Mild), GAD7 3M (Mild)	141.75	1	141.75	21.22	0.001	0.5	0.64
Gender (Mild)	1.82	1	1.82	0.38	0.55	0.01	0.03
RM Factor × Gender (Mild)	2.6	1	2.6	0.39	0.544	0.01	0.03
Residuals (Between Subjects)	57.79	12	4.82				
Residuals (Within Subjects)	80.15	12	6.68				
GAD7 BL (Moderate), GAD7 3M (Moderate)	528.4	1	528.4	39.39	<0.001	0.35	0.58
Gender (Moderate)	5.5	1	5.5	0.28	0.602	0	0.01
RM Factor × Gender (Moderate)	0.11	1	0.11	0.01	0.93	0	0
Residuals (Between Subjects)	575.46	29	19.84				
Residuals (Within Subjects)	388.99	29	13.41				
GAD7 BL (Severe), GAD7 3M (Severe)	3500.18	1	3500.18	162.53	<0.001	0.51	0.71
Gender (Severe)	6.13	1	6.13	0.21	0.646	0	0
RM Factor × Gender (Severe)	0.45	1	0.45	0.02	0.885	0	0
Residuals (Between Subjects)	1928.4	67	28.78				
Residuals (Within Subjects)	1442.87	67	21.54				
GAD7 BL (Average), GAD7 3M (Average)	3867.2	1	3867.2	195.61	<0.001	0.37	0.64
Gender (Average)	68.73	1	68.73	1.83	0.179	0.01	0.02
RM Factor × Gender (Average)	4.1	1	4.1	0.21	0.65	0	0
Residuals (Between Subjects)	4208.45	112	37.58				

**Table 4:** Comprehensive analysis of ANOVA repeated-measures testing outcomes.

Analysis Conducted:	GAD7 BL Mild	GAD7 3M Mild	GAD7 BL Moderate	GAD7 3M Moderate	GAD7 BL Severe	GAD7 3M Severe
Minimum	5.00	0.00	10.00	0.00	15.00	0.00
Maximum	9.00	10.00	14.00	19.00	21.00	21.00
Interquartile Range	2.00	4.00	2.50	8.00	3.00	10.00
Number of valid values	14.00	14.00	31.00	31.00	69.00	69.00
95% Confidence interval for mean	7.27–8.87	1.84–5.31	11.74–12.84	4.44–8.46	17.98–18.98	6.79–10.02
Mean ± Std.	8.07 ± 1.38	3.57 ± 3.01	12.29 ± 1.49	6.45 ± 5.49	18.48 ± 2.07	8.41 ± 6.74

**Table 5:** Age-specific demographic factors associated with GAD-7 scores collected. These factors were analyzed to range of patients enrolled in the trial.

Analysis Conducted:	Age (Mild)	Age (Moderate)	Age (Severe)	Age (Average)
Mean	52.07	50.06	46.2	47.97
Median	51	51	47	48.5
Mode	51	51	49	48
Minimum	31	27	19	19
Maximum	78	72	76	78
Interquartile Range	18.5	12.5	13	15
Number of valid values	14	31	69	114
Mean ± Std.	52.07 ± 13.23	50.06 ± 10.54	46.2 ± 10.73	47.97 ± 11.14

## Data Availability

The data that support the findings of this study are available from the corresponding author on request due to privacy issues in accordance with the consent provided by the participants. All data are freely accessible.
